# General Anesthesia Practices During the COVID-19 Pandemic in Turkey: A Cohort Study With a National Survey

**DOI:** 10.7759/cureus.10910

**Published:** 2020-10-12

**Authors:** Can Aksu, Sevim Cesur, Alparslan Kuş, Kamil Toker

**Affiliations:** 1 Anesthesiology, Kocaeli University, Kocaeli, TUR; 2 Anesthesiology, Kocaeli University School of Medicine, Kocaeli, TUR; 3 Anesthesiology and Reanimation, Kocaeli University, Kocaeli, TUR; 4 Anesthesiology and Reanimation, Istinye University, Istanbul, TUR

**Keywords:** covid-19, general anesthesia practice, video laryngoscope, personal protective equipment (ppe), positive pressure ventilation

## Abstract

Introduction

This study aimed to examine the anesthesia practices applied to the cases during the pandemic, to analyze the rate of the precautions taken in emergency/elective operations in non-COVID patients, what precautions were taken, what resources the clinics had, and the patient management in the perioperative period by organizing a survey among anesthesiologist in Turkey.

Methods

After obtaining approval from the Turkish Ministry of Health (2020-05-04T09_30_03) and the local ethics committee (GOKAEK-2020/10.09), a survey consisting of 21 questions was formed over the online survey inquiry (surveymonkey.com). The survey was conducted in Turkish.

Results

The survey aimed at reaching the anesthesiologists, who were Turkish Anesthesiology and Reanimation Society (TARD) members, by e-mail, and it was seen that 120 people out of approximately 2700 members who had received our e-mail participated in the survey. After the first case was reported in our country, it was understood that 62.1% of the participants stopped accepting elective cases in their institutions. The anesthesia method preferred in this period was general anesthesia by 47.6%, regional anesthesia by 52.1%, and sedation by 0.3%. The arrival time of coronavirus disease COVID-19 tests (PCR and/or rapid diagnostic kits showing antibodies) to the hospital was questioned; seven people (5.83%) stated that tests were not performed at their hospitals. It was observed that tests arrived and were applied at the hospitals of the remaining participants in an average of 2.7 ± 1.6 weeks. It was determined that 59.32% of the participants avoided positive pressure ventilation after induction, 5.98% of the intubation on the patients were performed by anesthesia technicians, 66.67% by anesthesiologists, 25.64% by senior resident doctors with at least two years of experience, and 1.71% by junior anesthesia assistants with less than two years of experience. The use of personal protective equipment (PPE) is applied by 95% of the participants. 22.69% of the participants stated that they preferred to use supraglottic airway (SGA) devices during this period. While 45.06% of the participants stated that they provided oxygen support to the patient with the mask belonging to the circuit after extubation, 14.8% preferred the nasal cannula, and 33.1% used an oxygen mask. Our results showed that 90% of additional precautions were taken in our country's clinics, and 95% of PPE was used. Also, the use of video laryngoscope (VL) was 75% in this period. Finally, it was found that 50.85% of the patients were taken to the recovery unit after being extubated, and 49.15% were sent directly to the service.

Conclusion

We can reveal that each clinic made arrangements according to its own conditions. We think that plans should be made to standardize clinical facilities and algorithms throughout the country. Apart from technological and financial facilities, we believe that the continuity of the training organized by national and international associations should be ensured so that anesthesiologists' knowledge, skills, and experience who manage this process can remain at the highest level.

## Introduction

The coronavirus outbreak, which began in Wuhan at the end of 2019, spread to the world gradually and was declared a "Public Health Emergency of International Concern" by the World Health Organization (WHO) as of January 2020. Due to the extent of the epidemic, the WHO declared the current situation a "global pandemic" on March 11, 2020 [1]. In Turkey, the first case was reported by the Ministry of Health on the same day, and precautions started to be taken for the pandemic. The necessary measures, such as starting pandemic hospitals, canceling elective surgical procedures, and suspension of health services by applying triage except for emergency cases, were taken to prevent the disease's spread.

Coronavirus disease (COVID-19) is a disease that spreads via the transmission of the virus in airway secretions through droplets and progresses to severe pneumonia. The severe acute respiratory syndrome coronavirus 2 (SARS-CoV-2) viral load is at the highest level in saliva, sputum, and airway secretions [2]. According to a systematic review conducted on the subject, airway interventions with the highest risk are listed as follows: tracheal intubation, tracheostomy, non-invasive ventilation, mask ventilation [3]. Intense aerosols are generated during the extubation of patients. As the disease spread, it is predicted that there are also many asymptomatic patients apart from asymptomatic patients and have been diagnosed. Some of these asymptomatic patients present to healthcare facilities for emergency surgery. Anesthesiologists are the professionals exposed at the highest risk since they also have to perform emergency surgeries such as cesarean section and trauma and provide airway management. Therefore, using personal protective equipment (PPE) and clinical planning are of vital importance in order to protect the anesthesia team and the entire team in the surgery.

The ministries of health, national and international anesthesia societies of countries have published many guidelines related to COVID-19 patients; however, it has been understood that each clinic manages the pandemic by making plans according to its facilities and patient profile. This study aimed to question anesthesia methods during the COVID-19 pandemic by organizing a survey among the anesthesiologists in Turkey. This study aimed to examine the anesthesia practices applied to the cases during the pandemic, to analyze the rate of the precautions taken in emergency/elective operations in non-COVID patients, what precautions were taken, what resources of the clinics had, and the patient management in the perioperative period.

## Materials and methods

After obtaining approval from the Ministry of Health (2020-05-04T09_30_03) and the local ethics committee (GOKAEK-2020/10.09), a survey consisting of 21 questions was formed over the online survey inquiry (surveymonkey.com). The survey inquiry was arranged so that the same person would be prevented from entering the survey multiple times. The survey was directed to the members of the Turkish Anesthesiology and Reanimation Society (TARD) via e-mail for the first time on May 12, 2020. It was published as an announcement on the website of the association, as well. The survey, which was shared again four weeks later via social media (like Whatsapp, Facebook, etc.), was closed at the end of the seventh week. In the e-mail sent to the participants together with the survey link, the purpose of the survey was clearly explained, and it was stated that participation in the survey was voluntary, and the participants could participate anonymously. The survey was conducted in Turkish. 

In the survey, only age, institution, and duration of anesthesia specialty were questioned as demographic data. Apart from these, the preoperative preparation phase, the use of PPE, the condition of the available equipment, the practices in the perioperative period, and the measures taken in the postoperative period were questioned (Appendices-1). Questions were prepared in the form of yes/no boxes, enabling them to choose the most appropriate and/or suitable ones among a few alternatives and fill the gaps.

The data obtained from the study were electronically recorded, and calculations were made by using the Statistical Packages for the Social Sciences (SPSS), Version 21 program (IBM, Armonk, NY). The mean, standard deviation, minimum and maximum values, and percentages of the data were calculated. 

## Results

The survey aimed to reach the anesthesiologists, who were TARD members, by e-mail, and it was seen that 120 people out of approximately 2700 members who were sent e-mail participated in the survey. The ages of the participants ranged between 25 and 64; (mean 44.6 ± 8 years). Their professional experiences were varied between 6 months and 38 years (mean 13.2 ± 7.9 years). It is understood that 33.61% of the participants received their specialization training at the Ministry of Health Training and Research Hospital, 63.03% at the Public University Hospital, and 3.36% from the Private University Hospital. The institutions where they are currently working as professionals are as follows; 20.17% state hospitals, 20.17% SBEAH, 31.93% university hospitals, and 27.73% private hospitals.

**Figure 1:**
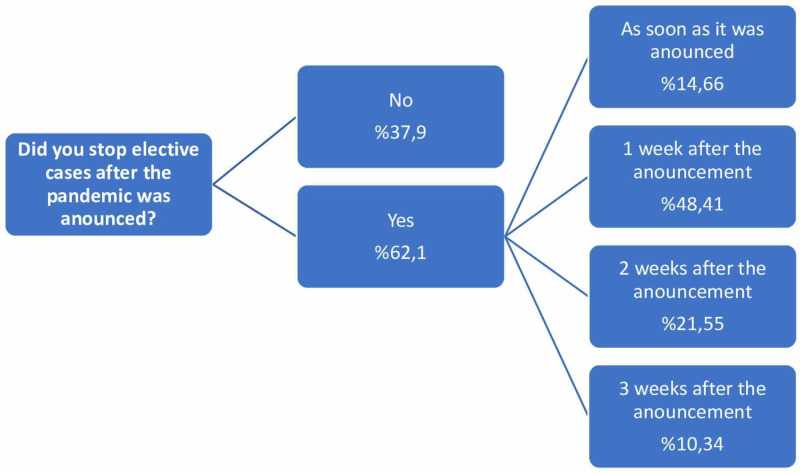
Elective cases during pandemic

After the first case was reported in our country, it was understood that 62.1% of the participants stopped accepting elective cases in their institutions, albeit at different timings (Figure 1). The rates of being COVID +/- or suspicious regarding the elective cases accepted to the hospital are 10.1%, 65.2%, and 24.7%, respectively. The arrival time of COVID-19 tests (PCR and/or rapid diagnostic kits showing antibodies) to the hospital was questioned; seven people (5.83%) stated that tests were not performed at their hospitals. It was observed that tests arrived and were applied at the hospitals of the remaining participants in an average of 2.7 ± 1.6 weeks. However, it was determined that the routine use of the tests remained at a low rate by 17.24% and that 82.76% were used only in selected cases.

 

The anesthesia method preferred in this period was general anesthesia by 47.6%, regional anesthesia by 52.1%, and sedation by 0.3%. 

**Figure 2:**
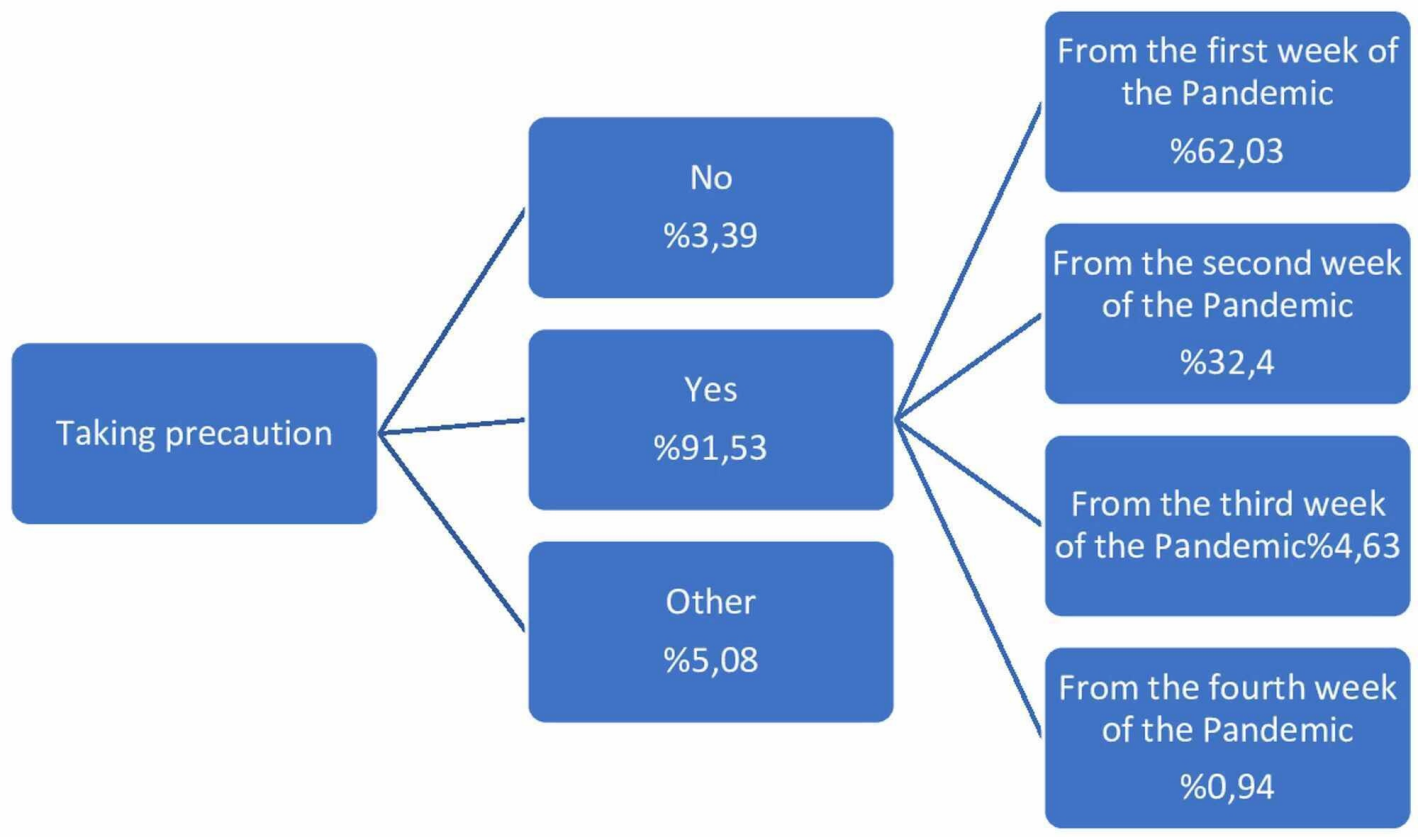
Precautions for COVID (-) patients Participants who answered "Other" stated that no cases were taken in their hospital

After the first case was announced, it was understood that the outbreak-related measures began to be taken at different times by 91.2%. The data regarding these times are shown in Figure 2.

**Figure 3:**
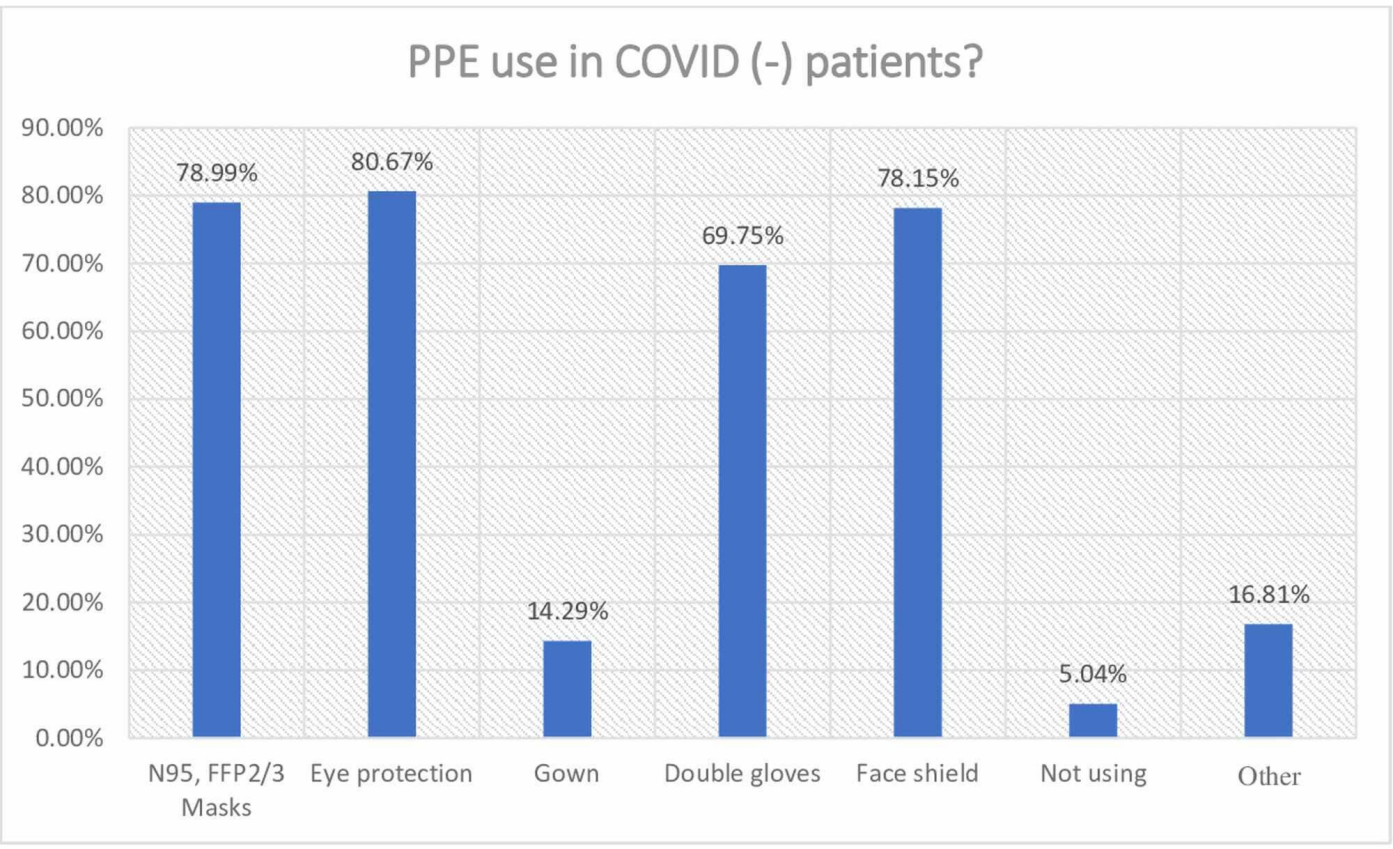
Personal protective equipment use PPE: personal protective equipment

The use of PPE is applied by 95% of the participants, albeit with different equipment (Figure 3). 5% of the participants stated that they did not use PPE. 

**Figure 4:**
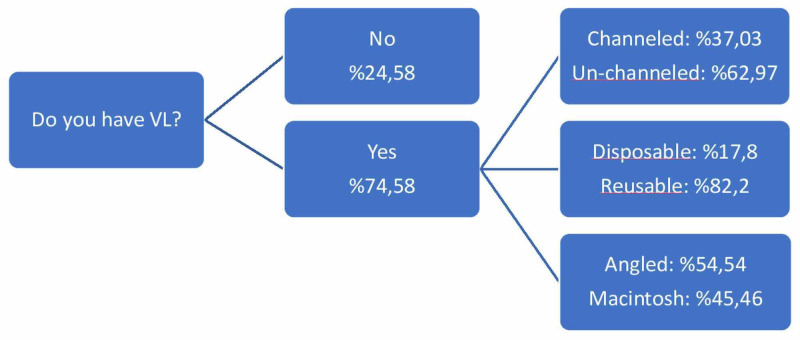
Video laryngoscope use and types VL: video laryngoscope

The answer to the question regarding whether there was a video laryngoscope in the clinics (VL), and, if there was, its features are seen in Figure 4. 

It was determined that 59.32% of the participants avoided positive pressure ventilation after induction, 5.98% of the intubation on the patients were performed by anesthesia technicians, 66.67% by anesthesiologists, 25.64% by senior resident doctors of two years or more, and 1.71% by junior anesthesia assistants of two years or less.

**Figure 5:**
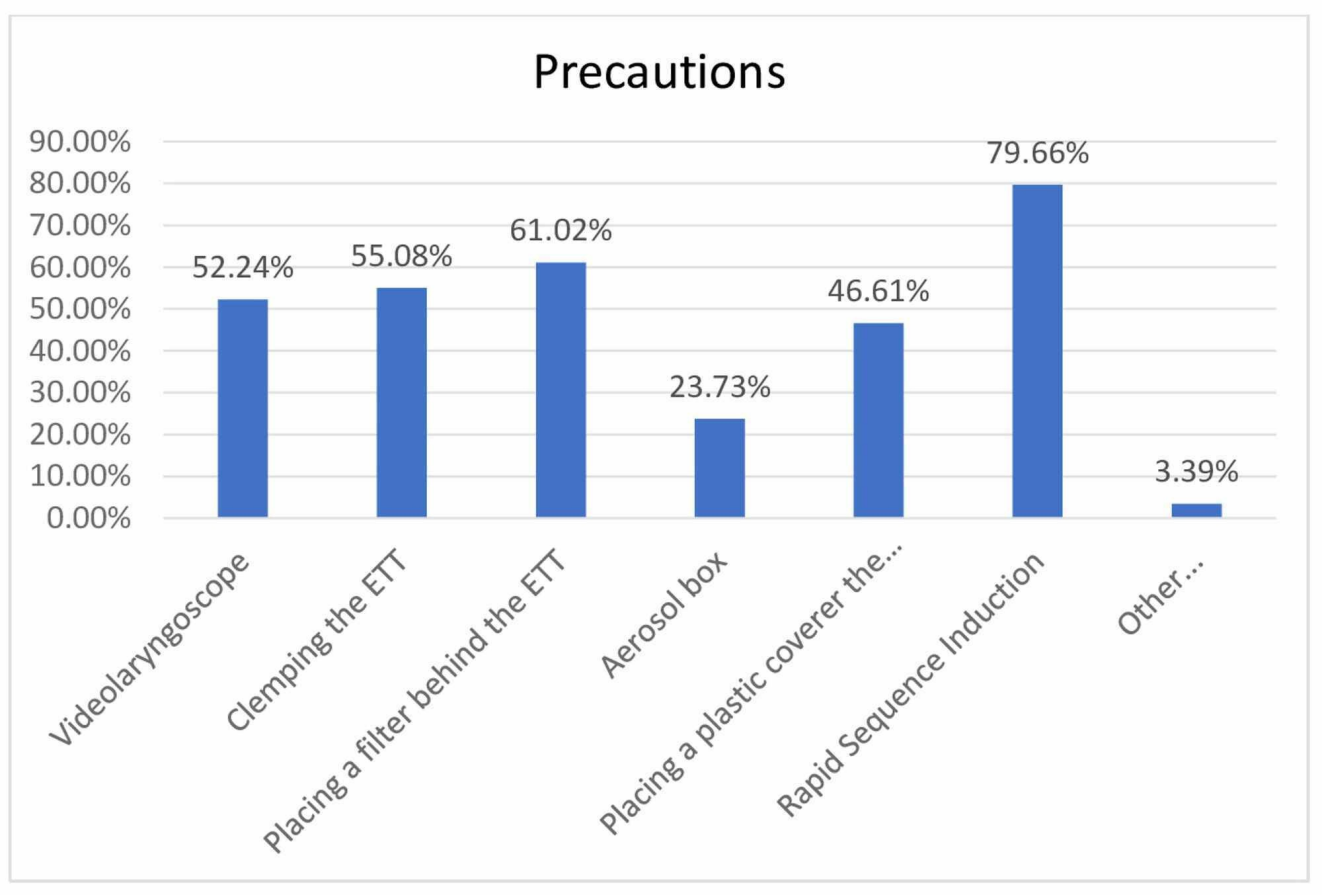
Precautions for intubations during the pandemic ETT: endotracheal tube

Additional precautions are taken while intubating patients are also shown in Figure 5. Considering the answers of the participants who marked the other option, it was seen that one person stated they did not take any precautions while others stated there were precautions such as precurarization, preoxygenation, and covering the laryngoscope blade with gloves.

22.69% of the participants stated that they preferred to use supraglottic airway (SGA) devices during this period, while 77.31% stated that they did not use them.

Additional measures taken during the extubation phase of the patients were found in Table 1. When the "other" answers in this question are examined, it is seen that one person did not take any measures, and the remaining responses included the use of sugammadex, the application of a surgical mask, implementation of the precautions taken during intubation and clamping the tube.

**Table 1:** Precautions during extubation

Aerosol Box	25,22%
Plastic covers over the patient	64,35%
Putting wet gauze on the patient's face	27,83%
Extubation without any aspiration	57,39%
Other	8,70%

While 45.06% of the participants stated that they provided oxygen support to the patient with the mask belonging to the circuit after extubation, 14.8% preferred the nasal cannula, and 33.1% used an oxygen mask. The participants who chose the "Other" option stated that they used an oxygen mask but covered it with a surgical mask or transparent plastic cover over or under it. However, this situation was also questioned, and it was found that the surgical mask or cover was used on the nasal cannula or mask with a rate of 81.2%.

Finally, it was found that 50.85% of the patients were taken to the recovery unit after being extubated, and 49.15% were sent directly to the service.

## Discussion

After the announcement of the pandemic by the WHO, the suspension of elective cases was recommended within the scope of the first precautions to be taken [4]. According to the results obtained from this survey, hospitals in Turkey suspended nearly 62% of elective cases; and it was observed that this precaution was implemented in the first week at approximately 63% of these hospitals. If the distribution of the participants by province or hospital characteristics is examined, it is seen that a significant part of the hospitals where elective surgical cases are not suspended are private hospitals; another part is the hospitals in provinces where COVID-19 cases are rarely seen. But this could also be a potential bias as these hospitals might have performed fewer tests.

In a study modeled to reflect 190 countries around the world, it was found that during the 12 weeks when the COVID-19 pandemic peaked in terms of the number of patients, approximately 28.404.603 cases were suspended; and this number has been reported to be approximately 72% of the cases worldwide [4]. In the modeling for Turkey, 82.000 cases were seen to be suspended per week [4]. According to this result, in countries like the United States, China, Russia, where the population is relatively high, Turkey has been determined as the seventh country identified with the most suspended elective cases numerically. However, according to our results, the proportionally less suspension of elective cases (63% vs. 72%) increases the risk of infection for anesthesiologists and surgical teams in our country.

The use of COVID-19 diagnostic tests routinely in every surgical patient was initially at a low rate (17%), and approximately 35% of these patients appeared to be COVID + or suspected. As a reason for not applying the tests routinely, as discussed in another question, it can be suggested that diagnostic tests were available across the country approximately in the third week of the pandemic. Other reasons may be the costs, late results of available diagnostic tests, low reliability, contact history, presence of symptoms, and the use of thoracic tomography for diagnosis in clinical approach.

It is known that airway interventions increase the risk of COVID by 6.6 times in the doctors who apply them [3]. Accordingly, the guideline, published by the American Society of Regional Anesthesia and Pain Medicine (ASRA) and European Society of Regional Anesthesia and Pain Therapy (ESRA), recommends applying regional techniques in every possible case. This guideline states that performing the surgery of patients under regional anesthesia with a correct triage will reduce the aerosol formation and decrease the need for intubation; therefore, the risk of contamination will also decrease [5]. According to our survey results, it is seen that regional anesthesia was preferred in approximately 52% of the cases across the country. The main reason for this could be that cases presenting during this period can be performed with neuraxial anesthesia and peripheral nerve blocks (PNB); such as extremity trauma, cesarean, and abdominal surgeries.

When the precautions taken in the cases operated during the pandemic were questioned, it was found that a vast majority (91.5%) of the precautions were taken. It is known that asymptomatic COVID (+) patients increase with the spread of the pandemic in the country. As we stated in our results, considering that COVID-19 tests are rarely applied in routine applications, it is a clinically correct approach to take precautions in every patient at a high rate. In a study conducted in February in China, the first country where the pandemic started, the rate of infection of healthcare workers was reported as 3.8%, while in Italy, one of the countries where the pandemic was the most severe, this rate was reported as 11% in April [6,7]. In our country, it is seen that this rate is 6.3%, according to the data published by the Turkish Ministry of Health on April 29. This study showed that, 90% of additional precautions were taken in our country's clinics, and 95% of PPE was used during this period. This could be the major reason that this rate was lower in our country.

When the precautions taken were examined, it was observed that protective glasses were used at the highest rate (80%), and this was followed by N95 masks (78%), face shields (78%), and double-layer gloves (69%), respectively. There are unfavorable conditions such as pressure on the face, wound on the nose, and breathing difficulties due to long-term use of masks. We think that the reason why the use of glasses is more than the use of masks is that these and similar difficulties are not encountered when using glasses; therefore, they are more easily tolerated. While the use of overalls was found to be the least in this group of patients without COVID-19 suspicion, 5% of the participants stated that they did not take any personal protective measures. During the applications in the pandemic period, we can only explain the lack of precautions taken by anesthesiologists with the lack of equipment; otherwise, there is no valid and logical explanation for keeping personal protection in the background during this period. As another reason, although it may be thought that there may be difficulty in reaching the equipment quickly in emergency cases, it is known that there is no emergency that neglects the use of PPE during the pandemic. 

During the pandemic, it is recommended that the performance of intubation should be done by the senior/most experienced person, and using a video laryngoscope is also recommended [8]. Although the use of VL is related to clinical facilities and the knowledge and skills of the anesthesiologist, it is seen that approximately 75% of the participants in the survey have and use VL in their clinic. In line with the recommendations, it was seen that senior practitioners performed the intubations at a very high rate (approximately 92%). It is known that VL prolongs the intubation time but increases the successful intubation rate in the first attempt. The use of VL is recommended by the consensus guidelines along with intubation boxes to keep the droplet spread at a minimum level [9]. However, it should be kept in mind that the use of boxes has its own difficulties. The most important of these is that it is challenging to operate from the box. Other concerns include that these boxes may not adequately prevent droplet spreading, and, as they are used repeatedly, adequate cleaning may not be achieved. Although VL features were questioned in our survey, it was not asked whether the screen was separate or integrated; however, it is stated that when using a VL with a separate screen, the distance between the patient and the practitioner will increase, and the risk of infection will decrease [9].

When other precautions taken in airway management are questioned, in parallel with the recommendations in the airway management of COVID-19 + patients, it is seen that the most common precautions are fast serial intubation, inserting a filter behind the tube, and clamping the tube, respectively. In this way, it can be stated that droplet formation and possible contamination through droplets are prevented.

Post-induction positive pressure ventilation practices of the participants were questioned; while 60% stated that they did not apply, 40% stated they did. However, the application of positive pressure ventilation after intubation is not recommended due to the formation of aerosol, and it is stated that it carries a high risk for the infection [10]. In a study that presents views on anesthesia management regarding pediatric patients, it is recommended to perform positive pressure ventilation at low pressures such as 10-12 cmH2O [11]. In the airway management guide published jointly by the associations, it is stated that mask ventilation should be applied only if necessary; when applied, it should be with two people, 2-hand mask ventilation technique, low flow, and low pressures [9].

The use of SGA in airway management was questioned in our survey, and it was observed that 77% of the participants did not use it. It is stated that intubation is safer than SGA, and it is recommended not to use SGA except for mandatory cases. It is seen that the general trend in our country is parallel to this. If a difficulty has been encountered in providing an airway; as an alternative to mask ventilation for leakage prevention, it is stated that second-generation SGA can be used [9]. When SGA is used, it has been reported that spontaneous ventilation may be preferred over-controlled ventilation to reduce leakage [9].

In our survey, the measures taken during extubation were also questioned, and it was seen that the most common one among them was covering the patient. Apart from this, the answers given are extubation without disconnecting the circuit, applying wet gauze on the patient's face, and extubation in the intubation box. It is stated that iv. lidocaine and/or dexmedetomidine can be administered to reduce cough. Another remarkable suggestion is SGA insertion by extubating the patient during deep anesthesia at the end of the operation and awakening the patient with SGA [9].

Following the extubation, providing oxygen support with the lowest possible flow (max 5L/min) to patients with a nasal cannula and placing a surgical mask over the nasal cannula are among the recommendations for the postoperative recovery period [9]. When our results are examined, it is seen that approximately half of the participants prefer using the mask belonging to the circuit. It has been noted that the use of an oxygen mask at a rate of 35% is preferred, and the nasal cannula is the least preferred. Habits during routine practices could be an explanation for these rates. However, if a nasal cannula/mask is used, 81% of the participants stated that they wear a surgical mask as a precaution.

In the postoperative period, it is recommended that COVID-19 patients be awakened in the operating room and transferred directly to the isolated room where they are staying [1]. However, there is no recommendation for other patients. This was finally questioned in the questionnaire, and it was seen that half of the participants sent patients directly to the ward, and half of them took the patients to the recovery room and then sent them to their ward. In patients taken to the recovery room according to clinical facilities, the risk of contamination can be reduced by taking additional precautions such as paying attention to the distance between the patients, taking oxygen support with the necessary minimum O2 flow, applying a surgical mask on the mask/nasal cannula.

Although this study has fulfilled its objective, it has some limitations. Firstly, since the surveys were sent to individuals, not institutions, they might have been answered by people working in the same institution, and the practices of the same clinic may be represented several times. Also, the strategy used by a single anesthesiologist does not reflect the strategy of the whole clinic and this could be a possible bias. Another limitation is that, given the number of anesthesiologists across the country, the survey was answered by a low percentage of participants. The reason can be thought to be the lack of spare time due to the intense workload of anesthesiologists during the pandemic period.

## Conclusions

Our results showed that 90% of additional precautions were taken in our country's clinics, and 95% of PPE was used. Also, the use of VL was 75% in this period. Based on these results, we can reveal that each clinic made arrangements according to its own conditions. Nevertheless, plans should be made to standardize clinical facilities and the development of standard algorithms throughout the country. Another option is that after the clinics' facilities are determined, and those facilities are categorized, algorithms with 2-3 options can be created accordingly. Another critical point revealed in our survey is the insufficient use of diagnostic tests. However, as much as the use of PPE is vital in this process, identifying and knowing patients is as important as preventing healthcare personnel from contamination. Apart from technological and financial facilities, the continuity of the training organized by national and international associations should be ensured so that the knowledge, skills, and experience of anesthesiologists who manage this process can remain at the highest level.

## Appendices

1. Age:.…….

2. Year of Experience:.……..

3. Training Hospital

( ) Ministry of Health Training and Research Hospital

( ) Public University Hospital

( ) Private University Hospital

4. Working Hospital 

( ) State Hospital

( ) Ministry of Health Training and Research Hospital

( ) University Hospital

( ) Private Hospital

5. Did you stop elective cases after the pandemic was announced?

( )      Yes

( )     No

6. When did you stop the elective cases? 

( ) As soon as it was announced

( ) 1 week after the announcement

( ) 2 weeks after the announcement

( ) 3 weeks after the announcement

( ) Other (Please specify):

7. Please classify the percentages of surgical cases referring to COVID +/- or suspected.

Covid (+) %.........

Covid (-) %.........

Suspected %.........

8. Do you test every patient for COVID before surgery? 

( )      Yes            ( )      No

9. When did your hospital be able to test patients for COVID?

 ( ) …………

10. Do you prefer general anesthesia or regional anesthesia for elective or emergency surgeries? Please indicate the percentages.

General Anesthesia %......

Regional Anesthesia %......

Other (Please Specify) %

11. Do you take precautions in COVID (-) patients? When did you start taking precautions?

( ) From the first week of the Pandemic

( ) From the second week of the Pandemic

( ) From the third week of the Pandemic

( ) From the fourth week of the Pandemic

( ) We do not take any precautions

( ) Other (Please specify)

12. Do you have a video laryngoscope in your clinic? Please specify it's properties.

( ) Yes

( ) No 

( ) Channeled   

( )  Un-channeled

   ( )  Disposable      

   ( )  Reusable

   ( )  Angled   

   ( )  Macintosh

13. Do you use personal protecting equipment (PPE) in COVID (-) patients?

( ) N95/FFP2-3 masks

( ) Gown

( ) Face shield/masks

( ) Eye protection

( ) Double gloves

( ) I do not use any PPE

14. Do you give positive pressure ventilation to COVID (-) patients after the induction?

( ) Yes

( ) No

15. Which of the following precautions do you use while intubating a COVID (-) patient?

( ) Videolaryngoscope

( ) Clemping the ETT

( ) Placing a filter behind the ETT

( ) Aerosol box

( ) Placing a plastic coverer the patient

( ) Rapid Sequence Induction

( ) Other (Please specify) …………………..

16. Who is doing the intubations?

( ) Anesthesia Nurse

( )  Specialists

( ) Residents (over 2 years of experience)

( ) Residents (under 2 years of experience)

17. Do you use supraglottic airway devices?

( ) Yes            ( ) No

18. Which of the following precautions do you take when extubating the patients?

( ) Aerosol box

( ) Plastic covers over the patient

( ) Putting wet gauze on the patient's face

( ) Extubation without any aspiration

19. How do you oxygenate the patient after extubation?

( ) With the facemask of the circuit

( ) With a nasal cannula

( ) With an oxygen mask

( ) Other (Please specify) ………

20. If you use nasal cannula / oxygen mask do you place a surgical mask over it?

( ) Yes

( ) No

21. Do you take patients to postoperative care unit or do you send them directly to the ward?

( ) Postoperative care unit

( ) Ward
